# Neuroinflammatory Biomarkers in Chronic Low Back Pain: Mechanisms, Clinical Evidence, and Translational Challenges

**DOI:** 10.3390/biomedicines14030557

**Published:** 2026-02-28

**Authors:** João Pinheiro, Pedro Lima, Ricardo Pestana, Miriam Sousa, José Alves, Hugo Ribeiro, Gonçalo Neto D’Almeida, Isabel Santana

**Affiliations:** 1 Neurosurgery Department, Hospital Dr. Nélio Mendonça, SESARAM, EPERAM, 9000-177 Funchal, Portugal; pedrolima@sesaram.pt (P.L.); ricardo@sesaram.pt (R.P.); 2 Faculty of Medicine, University of Coimbra, 3004-531 Coimbra, Portugal; hribeiroff@gmail.com (H.R.); isabeljsantana@gmail.com (I.S.); 3 Clinical Pathology Department, Hospital Dr. Nélio Mendonça, SESARAM, EPERAM, 9000-177 Funchal, Portugal; miriam.goncalves.sousa@gmail.com (M.S.); jose.alves@sesaram.pt (J.A.); 4 Group of Environment, Genetics and Oncobiology (CIMAGO), FMUC, Coimbra Institute for Clinical and Biomedical Research (iCBR), 3000-548 Coimbra, Portugal; 5 Neurosurgery Department, Unidade Local de Saude do Algarve, 8000-386 Faro, Portugal; neto.almeida@nms.unl.pt; 6 NOVA Medical School, 1169-056 Lisboa, Portugal

**Keywords:** biomarkers, central sensitization, chronic low back pain, glial activation, neuroinflammation

## Abstract

**Background:** Chronic low back pain (CLBP) is a leading cause of disability worldwide and remains clinically challenging due to its marked heterogeneity and limited correlation between structural pathology and symptoms. Increasing evidence suggests that neuroinflammatory mechanisms and central sensitization (CS) contribute to pain persistence in a clinically relevant subset of patients. This narrative review critically evaluates the current evidence on neuroinflammatory biomarkers in CLBP and discusses their translational potential for mechanism-based patient stratification. **Methods:** A comprehensive literature search was conducted in PubMed, Scopus, and Google Scholar using terms related to neuroinflammation, biomarkers, CLBP, CS, and glial activation. Studies were preferentially selected according to the following hierarchical criteria: (1) human studies directly investigating neuroinflammatory biomarkers in CLBP; (2) mechanistic human imaging or cerebrospinal fluid studies; (3) translational preclinical investigations providing direct mechanistic relevance; and (4) high-quality systematic reviews providing synthesis of biomarker evidence. As this was a narrative review, study selection was guided by conceptual relevance and translational significance rather than by formal systematic review methodology. **Results:** Converging evidence supports the involvement of neuroinflammatory processes in subgroups of patients with CLBP. In vivo TSPO-PET imaging and experimental data support glial activation in pain-processing regions. Cerebrospinal fluid studies report elevated chemokines, particularly interleukin-8 and monocyte chemoattractant protein-1, highlighting periphery-to-central nervous system inflammatory cross-talk and the concept of compartmentalized neuroinflammation. In parallel, epigenetic markers such as brain-derived neurotrophic factor DNA methylation have emerged as indirect correlates of CS-related pain phenotypes. In contrast, traditional systemic inflammatory markers show inconsistent and nonspecific associations. **Conclusions:** Neuroinflammatory biomarkers hold promise for mechanism-based stratification of CLBP, particularly for identifying patients with CS-driven pain. However, major methodological and translational challenges remain, including lack of standardization, limited accessibility of central nervous system-compartment measures, and the need for longitudinal and interventional validation. Future research should prioritize multi-marker and multi-compartment approaches integrated with functional phenotyping to establish clinical utility.

## 1. Introduction

Chronic low back pain (CLBP) is the leading cause of years lived with disability worldwide, affecting an estimated 568 million individuals [1]. Despite its substantial global burden, therapeutic outcomes remain disappointing, with many patients experiencing persistent or recurrent symptoms [2]. A key challenge lies in the marked heterogeneity of CLBP, which encompasses diverse underlying mechanisms that are insufficiently captured by current diagnostic classification systems [3,4].

CLBP, defined as pain persisting for more than 12 weeks, represents a heterogeneous clinical condition encompassing multiple biological mechanisms and clinical presentations. Patients may present with pain in the absence of a clearly identifiable structural lesion (commonly termed nonspecific CLBP) or with degenerative lumbar conditions such as disc degeneration, in which structural abnormalities coexist with complex neurobiological pain processes. Although epidemiological studies estimate that a large proportion of cases are classified as nonspecific, this designation primarily reflects current diagnostic limitations rather than a true absence of biological pathology [2,5]. Consistent with this complexity, the correlation between radiographic abnormalities and pain severity or disability remains weak despite the central role traditionally attributed to imaging in clinical evaluation [6].

Over the past two decades, a major conceptual shift has occurred with the recognition that chronic pain often reflects maladaptive neuroplastic changes within the central nervous system (CNS), rather than ongoing peripheral tissue damage [7]. Central sensitization (CS), defined as increased responsiveness of pain-processing neurons in the CNS to normal or subthreshold inputs, has been identified as a key mechanism driving symptom persistence in a clinically relevant subset of patients with CLBP [8,9].

Neuroinflammation is increasingly recognized as an important contributor to the development and maintenance of CS [10]. Preclinical studies demonstrate that sustained nociceptive input can activate spinal and supraspinal glia, particularly microglia and astrocytes, which release pro-inflammatory mediators that amplify nociceptive transmission and promote neuronal hyperexcitability [11,12]. This neuroimmune crosstalk establishes a self-sustaining inflammatory microenvironment that may persist well beyond the resolution of the initial peripheral insult [13,14].

These insights carry important clinical implications. If biologically distinct subgroups exist within the broader CLBP population, biomarker-based stratification may enable more individualized and mechanism-oriented treatment strategies [4,15]. Neuroinflammatory biomarkers, in particular, may help identify patients whose symptoms are predominantly driven by CS related mechanisms [3]. This perspective is increasingly reflected in recent international initiatives and consensus statements advocating phenotype-driven and biomarker-informed approaches to low back pain and chronic pain more broadly [16,17].

Accordingly, this narrative review critically evaluates the current evidence on neuroinflammatory biomarkers in CLBP. We focus on three major categories: (1) glial-related biomarkers, including glial fibrillary acidic protein (GFAP) and translocator protein (TSPO) positron emission tomography (PET) imaging; (2) chemokines and neuroimmune signaling molecules such as monocyte chemoattractant protein-1 (MCP-1) and interleukin-8 (IL-8); and (3) emerging epigenetic biomarkers, including deoxyribonucleic acid (DNA) methylation patterns of brain-derived neurotrophic factor (BDNF). We examine their biological relevance, available human evidence, and translational challenges. Finally, we discuss why traditional systemic inflammatory markers have shown inconsistent clinical utility, emphasizing the concept of compartmentalized neuroinflammation.

While this review focuses on CLBP, it is important to recognize that a substantial portion of the available human biomarker literature derives from cohorts with degenerative disc disease (DDD) or lumbar disc herniation (LDH). Because access to cerebrospinal fluid (CSF), surgical tissue, and advanced neuroimaging data is often limited to clinically indicated populations, these cohorts provide much of the direct human evidence available for studying neuroinflammatory mechanisms. Accordingly, studies involving DDD or LDH populations are included as translational human models informing mechanisms relevant to chronic lumbar pain persistence. Findings from these cohorts are interpreted cautiously, with emphasis placed on mechanistic insights rather than disease-specific generalization to all CLBP phenotypes.

## 2. Methods

This narrative review was based on a structured literature search designed to identify studies investigating neuroinflammatory biomarkers in CLBP and closely related degenerative lumbar conditions. Searches were conducted in PubMed and Scopus as primary databases. Google Scholar was included as a supplementary source to capture interdisciplinary research (e.g., neuroimaging, epigenetics, and systems biology) and recently published articles that may not yet have been indexed in PubMed or Scopus, as well as to facilitate citation tracking of key publications.

The search covered publications from January 2000 to December 2025, with the final search performed on 5 December 2025. Foundational mechanistic and conceptual studies published before 2015 were included when necessary to provide historical and mechanistic context, while greater emphasis was placed on human and translational literature published from 2015 onward.

Search terms were combined using Boolean operators and included variations in the following concept domains: (1) condition-related terms (“chronic low back pain,” “low back pain,” “lumbar pain”); (2) mechanistic terms (“neuroinflammation,” “central sensitization,” “nociplastic pain”); and (3) biomarker-related terms (“biomarker,” “cytokine,” “chemokine,” “glial activation,” “microglia,” “astrocyte,” “TSPO,” “GFAP,” “MCP-1,” “IL-8,” “BDNF,” and “epigenetics”). Reference lists of relevant articles and recent reviews were manually screened to identify additional studies of conceptual or translational relevance.

Studies were eligible if they examined molecular (blood, CSF, or tissue), imaging-based (e.g., TSPO-PET), or epigenetic biomarkers in adult patients with CLBP or closely related chronic lumbar degenerative conditions. Studies were also included if they provided mechanistic insight directly relevant to neuroimmune signaling and CS. Preclinical studies were selectively incorporated when they offered mechanistic context necessary to interpret human findings.

Studies focused exclusively on acute low back pain, purely structural imaging without mechanistic implications, non-inflammatory biomarkers, case reports without biomarker measurement, or non-English publications were excluded.

**Figure 1:**
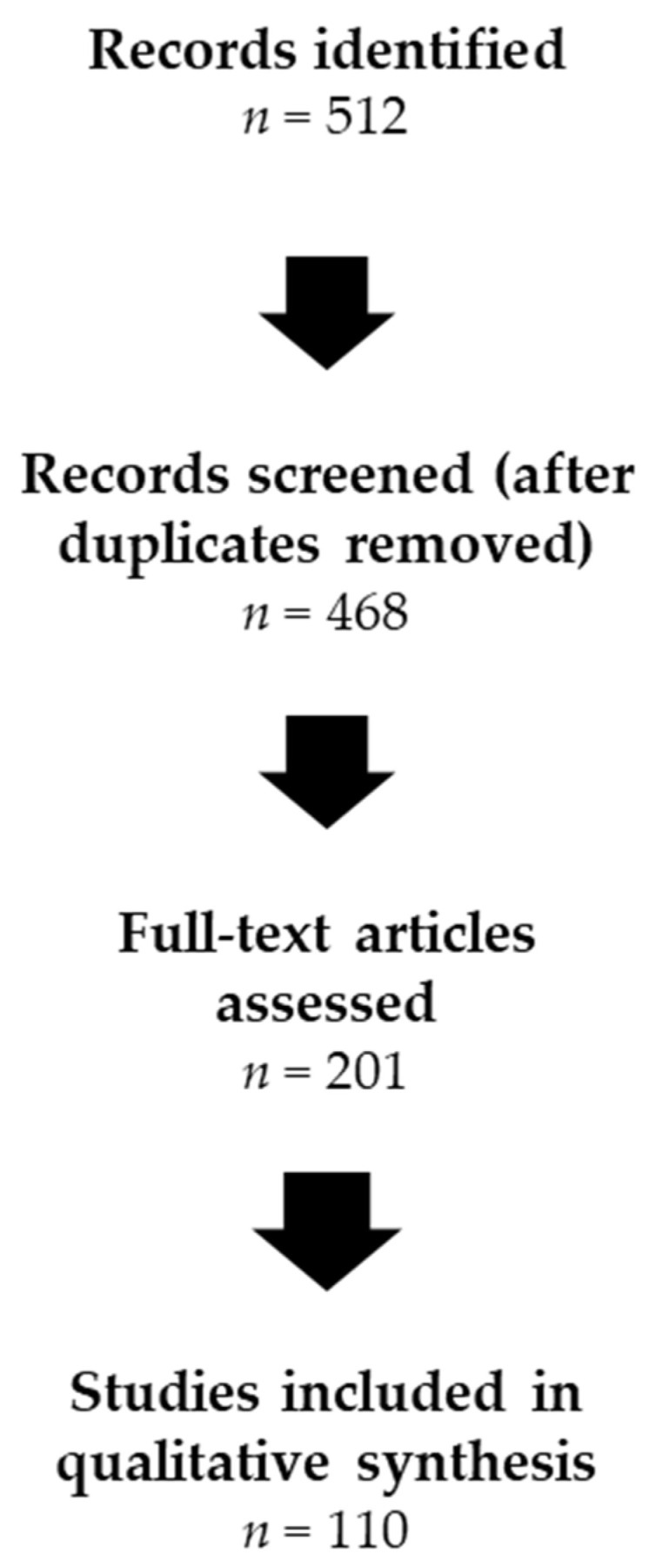
Structured flow diagram of literature identification and qualitative study selection for this narrative review.

The initial search yielded approximately 512 records. After title and abstract screening for relevance, 201 articles underwent full-text review. Of these, approximately 110 studies were considered most pertinent based on direct measurement of candidate neuroinflammatory biomarkers and/or explicit linkage to pain phenotypes or CS features, and informed the qualitative synthesis presented in this review. The study selection process is summarized in Figure 1.

Given the narrative and concept-driven nature of this manuscript, no formal risk-of-bias scoring was performed. Instead, the strength of evidence was qualitatively evaluated based on consistency across human studies, robustness of study design (including cohort size and longitudinal data), replication across independent cohorts, and proximity to potential clinical translation. The objective of this review was conceptual and translational synthesis rather than exhaustive systematic coverage or quantitative meta-analysis.

## 3. Pathophysiological Rationale: Neuroinflammation and Pain Chronicity

### 3.1. Central Sensitization in Chronic Low Back Pain

Much of the available human mechanistic evidence discussed in the following sections originates from degenerative lumbar pain conditions (e.g., LDH and DDD), which provide accessible clinical models for investigating neuroimmune mechanisms relevant to chronic pain persistence. Within this framework, CS has been proposed as a key mechanism underlying pain persistence in a subset of patients with CLBP, providing a physiological link between clinical symptoms and neuroimmune dysregulation. CS represents a state of increased excitability within central nociceptive pathways, characterized by amplified neural responses to normal or subthreshold stimuli [18]. The clinical hallmarks of CS include secondary hyperalgesia, allodynia, and enhanced temporal summation. Impaired descending pain modulation is also frequently observed, although this feature is typically identified through quantitative sensory testing (QST) rather than routine clinical examination [19]. In CLBP populations, these manifestations present as widespread pressure pain hypersensitivity and reduced pain thresholds at both local and remote sites, patterns that are incompatible with a purely peripheral nociceptive source [20,21].

QST studies provide robust objective evidence of CS in CLBP. Patients consistently demonstrate generalized hyperalgesia, with reduced pressure pain thresholds at both lumbar and distant anatomical regions [22]. Temporal summation of pain is also enhanced, reflecting increased excitability within dorsal horn neurons [23]. Complementing these findings, human neuroimaging studies have also demonstrated functional and neurochemical alterations within key nodes of the descending pain modulatory system, including dysregulated metabolite profiles in the periaqueductal gray in patients with CLBP [24].

Clinically, CS defines a distinct subgroup of patients with CLBP. Individuals with high CS profiles report greater pain intensity, more widespread pain distribution, higher disability, and poorer treatment outcomes [25]. Epidemiological estimates suggest that approximately 20–30% of CLBP patients exhibit clinically significant CS, indicating that a substantial proportion of patients belong to this centrally mediated pain subgroup [26].

The neurobiological mechanisms underlying CS involve changes in synaptic efficacy, altered inhibitory–excitatory balance, and sustained amplification of nociceptive processing. A growing body of evidence identifies neuroinflammation mediated through glial activation and immune-derived signaling molecules as a key driver of these neuroplastic changes [10,27]. These concepts align with contemporary models of nociplastic pain and provide the conceptual bridge to the neuroimmune pathways discussed in the following section.

### 3.2. Neuroimmune Crosstalk and Glial Activation

The traditional view of pain as a purely neuronal phenomenon has shifted with the recognition of the critical role played by glial cells, particularly microglia and astrocytes, in modulating nociceptive processing [14,28]. In the healthy CNS, glia provide structural support and regulate synaptic transmission. However, following peripheral injury or inflammation, glial cells undergo activation, and release inflammatory mediators that substantially alter pain signaling [29].

Microglia, the resident immune cells of the CNS, act as primary responders to tissue injury signals [30]. Peripheral tissue injury, inflammatory processes, or sustained nociceptive afferent input can trigger microglial activation in the spinal dorsal horn through multiple pathways, including purinergic signaling mediated by adenosine triphosphate release and P2X4 receptor activation, chemokine gradients, and damage-associated molecular patterns [31,32]. Activated microglia increase the expression of proteins associated with glial activation, including TSPO [33].

Once activated, microglia release a cascade of pro-inflammatory mediators that increase the excitability of neurons involved in spinal nociceptive processing. Key mediators include BDNF, which downregulates potassium-chloride cotransporter KCC2 and disrupts inhibitory neurotransmission; tumor necrosis factor-α (TNF-α); and interleukin-1β (IL-1β), which enhances neuronal excitability [34,35]. Through these mediators, glial activation shifts the balance between excitation and inhibition within spinal nociceptive circuits, lowers neuronal firing thresholds, and promotes activity-dependent synaptic potentiation, thereby stabilizing a hyperexcitable network state. This microglia–neuron crosstalk establishes a self-reinforcing positive-feedback loop that contributes to CS [36].

Astrocytes, the most abundant glial cells, play complementary roles in pain chronicity [37]. Following microglial activation, astrocytes undergo reactive gliosis, characterized by upregulation of GFAP [38]. Reactive astrocytes release pro-inflammatory cytokines, chemokines such as MCP-1, and glutamate, further amplifying neuronal hyperexcitability and perpetuating CS [39].

These central mechanisms do not operate in isolation. Increasing evidence indicates that peripheral inflammatory signals can directly influence and sustain CNS neuroinflammation. Neuroimmune crosstalk extends beyond local spinal mechanisms and encompass bidirectional communication between peripheral tissues, spinal cord, and brain [40]. In CLBP, degenerating intervertebral discs and inflamed facet joints release inflammatory mediators into the local microenvironment [41]. These peripheral signals can reach the CNS through multiple pathways, including retrograde axonal transport along sensory afferents, diffusion across the blood-spinal cord barrier, and systemic circulation [42].

Recent evidence demonstrates that periphery-to-CNS inflammatory cross-talk occurs in degenerative low back pain conditions. Rosenström et al. performed a comprehensive multi-compartment analysis in patients with LDH and DDD, measuring 92 inflammatory mediators in paired CSF and serum samples. They found significantly elevated IL-8 levels in the CSF of LDH patients, which correlated with pain intensity and spinal pain sensitivity in males. In addition, MCP-1 showed significant CSF–serum correlations in DDD patients, and serum MCP-1 levels were associated with spinal pressure pain sensitivity. Importantly, many inflammatory mediators elevated in CSF were not increased in serum, demonstrating compartmentalized neuroinflammation that cannot be captured by blood-based assessments alone [43].

Complementing these findings, Palada et al. analyzed gene expression in disc tissue from patients with degenerative disc disease and showed that TSPO expression correlated with back pain intensity, providing direct evidence linking peripheral disc inflammation to clinical symptom severity [44].

Together, these findings highlight the need to characterize specific neuroinflammatory biomarkers across compartments, which is addressed in the following section.

## 4. Neuroinflammatory Biomarkers in Chronic Low Back Pain

It is important to acknowledge that much of the direct human evidence for neuroinflammatory processes in CLBP has been derived from patients with degenerative lumbar conditions such as LDH and DDD, which represent common clinical causes of persistent lumbar pain rather than entities entirely distinct from CLBP. This primarily reflects practical and ethical constraints related to access to CSF, surgical tissue, and advanced neuroimaging data. Accordingly, these conditions are considered here as translational human models of peripheral–central neuroimmune interaction, and biomarker findings are interpreted as mechanistically informative across the spectrum of chronic lumbar pain phenotypes rather than exclusive to strictly defined non-specific CLBP populations.

In this section, we synthesize the available evidence on candidate neuroinflammatory biomarkers in CLBP, encompassing molecular, epigenetic, and imaging-based markers. In the context of chronic pain research, the term “biomarker” is used in a broad sense, referring not only to circulating or CSF molecules, but also to imaging markers and epigenetic signatures that reflect neuroimmune activity within the pain system. According to established biomarker qualification frameworks, including the FDA–NIH BEST (Biomarkers, EndpointS, and other Tools) classification, the candidates discussed in this review should primarily be interpreted as mechanistic and stratification biomarkers with potential diagnostic, prognostic, and monitoring applications, rather than validated predictive or treatment-guiding tools at the current stage of evidence.

Importantly, the level of evidence and translational maturity varies substantially across these candidates. Table 1 provides an overview of the main neuroinflammatory biomarkers investigated in CLBP, their biological compartments, the type of supporting evidence, and their current translational readiness. Among the available approaches, TSPO-PET currently represents the most direct in vivo marker of neuroinflammation in human CLBP, whereas CSF chemokines reflect compartment-specific molecular processes, and blood-based or epigenetic markers should still be regarded as indirect or exploratory surrogates.

This table summarizes the main candidate neuroinflammatory biomarkers investigated in CLBP, including molecular, epigenetic, and imaging-based markers. For each biomarker, the biological compartment, type of supporting evidence, studied populations, association with pain or CS, overall strength of evidence, and principal limitations are indicated. The table highlights the marked heterogeneity in evidence maturity across candidates and emphasizes the concept of compartmentalized neuroinflammation and the current translational gap between mechanistic relevance and clinical applicability.

### 4.1. Central Neuroinflammatory Biomarkers

#### 4.1.1. GFAP and TSPO as Markers of Glial Activation

GFAP is an intermediate filament protein expressed predominantly in astrocytes, whose expression is markedly upregulated during reactive astrogliosis, making it one of the most widely used markers of astrocyte activation [45,61]. In preclinical pain models, GFAP immunoreactivity is consistently increased in spinal dorsal horn astrocytes following peripheral nerve injury, and the magnitude of this response correlates with pain-related behaviors [62]. Reactive astrocytes influence nociceptive processing through multiple mechanisms, including the release of pro-inflammatory mediators such as IL-1β, TNF-α, and MCP-1, which contribute to dorsal horn neuronal hyperexcitability [62], as well as impaired glutamate clearance due to altered expression of astrocytic glutamate transporters, thereby facilitating N-Methyl-D-Aspartate (NMDA) receptor–mediated excitatory signaling [63].

Despite robust preclinical evidence, translation of GFAP as a clinical biomarker remains limited. Under physiological conditions, GFAP is largely confined to the CNS, and intact blood–brain barrier function restricts its presence in peripheral circulation. Although recent ultrasensitive immunoassay platforms allow reliable detection of serum GFAP in conditions associated with blood–brain barrier disruption [64], no studies to date have systematically evaluated circulating GFAP in CLBP populations. Accordingly, at present, GFAP should be regarded primarily as a mechanistic marker of astrocytic activation rather than a clinically validated biomarker in this context. Nevertheless, its strong mechanistic link to central neuroinflammatory processes and the increasing feasibility of reliable serum quantification support its consideration as a candidate translational biomarker for future studies in well-phenotyped CLBP cohorts.

While GFAP captures astrocytic reactivity, it does not reflect the full spectrum of glial involvement in CLBP. Microglial activation constitutes a parallel and potentially critical component of neuroimmune signaling implicated in chronic pain mechanisms [13,65]; however, validated microglia-specific circulating biomarkers in CLBP are currently lacking. Candidate molecules proposed in broader neuroinflammatory contexts include triggering receptor expressed on myeloid cells 2 (TREM2), particularly its soluble form (sTREM2), and YKL-40 (CHI3L1), both investigated as markers of microglial activation in human CNS disorders [66,67]. Importantly, when measured in peripheral blood, these markers lack specificity for central microglial activation and may reflect systemic myeloid activity. To date, no studies have systematically evaluated these candidate markers in well-phenotyped CLBP cohorts. Accordingly, their translational role in CLBP remains exploratory.

In contrast to molecular markers that require indirect inference, in vivo imaging approaches allow direct assessment of glial activity within the CNS. Among these, positron emission tomography (PET) using radioligands targeting the 18-kDa translocator protein (TSPO) represents the most extensively studied and, to date, the most direct in vivo approach to assess neuroinflammation in humans [46]. TSPO is a mitochondrial protein upregulated in activated microglia and reactive astrocytes and is widely used as an imaging marker of glial activation [47].

Converging TSPO-PET evidence supports the presence of neuroinflammatory activation in CLBP. Loggia et al. synthesized TSPO-PET findings across chronic pain conditions and reported elevated TSPO signal in several pain-processing regions, including the thalamus, primary somatosensory cortex, and primary motor cortex, in patients with CLBP compared with healthy controls [48]. Similarly, Shraim et al. demonstrated increased TSPO-PET signal in sensorimotor cortical regions of individuals with low back pain maintained by nociplastic mechanisms and showed that the degree of neuroinflammatory activation was associated with altered sensorimotor function, further supporting the clinical relevance of TSPO imaging in this population [49].

TSPO-PET evidence is not limited to supraspinal structures. Albrecht et al. demonstrated increased TSPO binding in the spinal cord and nerve roots of patients with chronic radicular pain due to disc herniation, indicating that glial activation within spinal nociceptive pathways can be detected in vivo in humans [50]. In addition, several studies have reported associations between TSPO signal intensity and clinical measures such as pain intensity, symptom duration, and functional disability [48,50] further supporting its potential pathophysiological relevance.

Despite representing the strongest currently available in vivo evidence of neuroinflammation in chronic pain, TSPO-PET has important limitations that preclude routine clinical use. Moreover, the current TSPO-PET evidence in CLBP remains based on relatively small and clinically heterogeneous cohorts and should be regarded as proof-of-concept rather than clinically established. From a practical perspective, TSPO-PET requires specialized PET infrastructure and radiochemistry, binding affinity varies across individuals due to TSPO genetic polymorphisms, and TSPO expression is not cell-type specific, being present in both microglia and astrocytes [46,47]. Therefore, TSPO-PET should currently be regarded as a powerful research tool rather than a scalable clinical biomarker.

#### 4.1.2. Epigenetic Biomarkers and BDNF-Related Mechanisms

BDNF plays a central role in pain neurobiology, acting both as a neurotrophin and as a key mediator of neuroimmune-driven pain amplification [68]. In the context of chronic pain, BDNF is a critical component of microglia–neuron crosstalk: activated microglia release BDNF, which binds to tropomyosin receptor kinase B receptors on dorsal horn neurons and induces downregulation of the potassium–chloride cotransporter KCC2 [69]. This disrupts chloride homeostasis, converting GABA-mediated inhibition into excitation, a fundamental mechanism of CS [54]. These well-established mechanistic roles provide a strong biological rationale for considering BDNF-related pathways in chronic pain, but do not, by themselves, establish BDNF as a direct biomarker of neuroinflammation in humans.

In addition to candidate gene-specific approaches, emerging evidence suggests that CLBP may be associated with broader epigenetic alterations in peripheral immune cells. Grégoire et al. performed an epigenome-wide analysis of isolated T cells in individuals with CLBP and identified sex-specific DNA methylation signatures capable of distinguishing patients from healthy controls [55]. These findings support the concept that immune-cell-specific epigenetic reprogramming may characterize CLBP at a systemic level. Complementing these observations, Aroke et al. reported differential DNA methylation patterns and pathway enrichment analyses implicating immune and inflammatory signaling networks in adults with non-specific CLBP [56]. While these studies remain exploratory and require replication in larger, well-phenotyped cohorts, they provide important preliminary evidence that immune-cell epigenetic signatures may reflect chronic pain–related neuroimmune interactions beyond single candidate genes.

Within this broader context of immune-cell epigenetic remodeling, BDNF has emerged as one of the most extensively investigated candidate genes linking epigenetic regulation to pain-related neuroplasticity. DNA methylation, a stable epigenetic modification regulating gene expression, represents one of the principal mechanisms through which BDNF activity may be modulated [70]. The BDNF gene contains multiple CpG islands susceptible to epigenetic modification, and changes in its methylation status have been implicated in chronic pain conditions [71,72].

Polli et al. conducted a landmark study investigating BDNF DNA methylation in patients with chronic fatigue syndrome and fibromyalgia, conditions characterized by widespread pain and CS features that overlap with CLBP. They measured BDNF methylation in peripheral blood leukocytes and assessed pain sensitivity using QST. The study showed a strong association between BDNF methylation and pain-related phenotypes. Patients with lower BDNF methylation, exhibited more widespread hyperalgesia, reduced pressure pain thresholds, and greater temporal summation of pain. Importantly, BDNF methylation levels correlated inversely with BDNF mRNA expression, supporting the functional relevance of this epigenetic marker [72].

Taken together, these findings support a molecular cascade linking epigenetic regulation to clinical pain phenotypes. Chronic stress or inflammatory states may promote reduced BDNF methylation, leading to increased BDNF expression and enhanced glia–neuron signaling within spinal nociceptive circuits, thereby contributing to CS and the development of widespread hyperalgesia [51]. From a practical perspective, the ability to measure BDNF methylation in peripheral blood makes this marker attractive as a research tool.

Paoloni-Giacobino et al. extended these observations to chronic musculoskeletal pain populations, including patients with CLBP, and reported that BDNF methylation levels varied according to biopsychosocial complexity, defined as a composite measure of pain severity, psychological distress, and functional impairment. Patients with high biopsychosocial complexity exhibited significantly lower BDNF methylation levels than those with low complexity [52]. These findings suggest that BDNF methylation may reflect not only neurobiological mechanisms of pain but also broader dimensions of the pain experience that integrate biological and psychosocial factors.

The integration of biological and psychosocial dimensions through epigenetic mechanisms is consistent with contemporary biopsychosocial models of chronic pain [73]. Psychological stress and adverse life events have been shown to induce epigenetic modifications in stress-responsive genes, including BDNF [71]. In the context of CLBP, this framework suggests that patients with a high psychosocial burden may develop epigenetic signatures that contribute to the development and persistence of CS [52,74].

Despite its conceptual appeal, BDNF methylation currently remains an indirect and exploratory biomarker. The available studies have been conducted primarily in chronic fatigue syndrome, fibromyalgia, or mixed musculoskeletal pain populations, and replication in well-characterized CLBP cohorts is required [52,72]. Moreover, BDNF methylation measured in peripheral blood may not faithfully reflect epigenetic regulation within CNS pain circuits [75]. Finally, the specificity of BDNF methylation for pain-related mechanisms, as opposed to broader stress-related or affective processes, remains to be established [73]. Accordingly, at present, BDNF methylation should be regarded as a promising research tool and a potential stratification marker, rather than a clinically validated biomarker of neuroinflammation.

### 4.2. Chemokines and Neuroimmune Signaling

#### 4.2.1. Monocyte Chemoattractant Protein-1 (MCP-1/CCL2)

MCP-1, also known as CCL2, is a chemokine that plays a central role in neuroimmune signaling and pain chronicity [76]. Its primary biological function is to recruit monocytes, macrophages, and other immune cells to sites of tissue injury through binding to its cognate receptor CCR2 [57]. Mechanistically, MCP-1 contributes to pain through multiple pathways. In peripheral tissues, MCP-1 is released by damaged cells and sensory neurons, creating chemotactic gradients that attract immune cells [58]. In the spinal cord, MCP-1 is produced by activated astrocytes and acts on microglial CCR2 receptors, promoting microglial activation and release of pro-inflammatory cytokines [62]. Additionally, MCP-1 exerts direct neuromodulatory effects by enhancing neuronal excitability and increasing NMDA receptor–mediated currents [77].

Preclinical studies have established a causal role for MCP-1 in pain chronicity. Genetic deletion of MCP-1 or its receptor CCR2, as well as pharmacological antagonism of CCR2, attenuates pain-related behaviors in models of neuropathic pain and disc puncture–induced back pain [58,59,78]. These data provide strong mechanistic support for the involvement of MCP-1 in neuroimmune-driven pain amplification.

In humans, however, the biomarker value of MCP-1 is highly dependent on the biological compartment in which it is measured. Systematic reviews have reported elevated serum or plasma MCP-1 levels in CLBP patients compared with healthy controls [79], and several studies have described correlations between circulating MCP-1 concentrations and clinical pain severity [43,80]. Nevertheless, these associations are heterogeneous and of variable effect size, and circulating MCP-1 lacks disease specificity, as elevated levels are also observed in cardiovascular disease, metabolic syndrome, and obesity [53].

The most compelling human evidence for a pathophysiologically relevant role of MCP-1 comes from multi-compartment studies examining CSF, serum, and tissue levels. Palada et al. measured MCP-1 in CSF and serum of patients with LDH and DDD and found significant CSF–serum correlations in DDD patients, suggesting coordinated peripheral–central inflammatory signaling. Notably, serum MCP-1 levels were associated with spinal pressure pain sensitivity in female DDD patients, indicating potential sex-specific biomarker–pain relationships [44].

Rosenström et al. extended these observations by performing high-dimensional inflammatory profiling of paired CSF and serum samples. Their analysis identified MCP-1 among the mediators showing coordinated regulation across compartments, further supporting the concept of periphery-to-CNS inflammatory cross-talk in degenerative low back pain conditions [43].

Importantly, the clinical heterogeneity of MCP-1 findings deserves emphasis. Not all patients with CLBP exhibit elevated MCP-1 levels, and reported effect sizes vary substantially across studies [81], likely reflecting the underlying mechanistic heterogeneity of CLBP. In addition, sex differences appear to modulate MCP-1–pain associations, as correlations with pain sensitivity were more consistent in females than in males in some cohorts [44], possibly reflecting sex-specific immune–pain interactions [82].

Taken together, these findings indicate that MCP-1 should not be regarded as a standalone or disease-specific biomarker of CLBP. Rather, its main value lies in its integration into multi-marker and multi-compartment biomarker panels aimed at capturing periphery-to-CNS inflammatory cross-talk in selected patient subgroups.

#### 4.2.2. Interleukin-8 (IL-8/CXCL8) and Periphery-to-CNS Cross-Talk

Interleukin-8 (IL-8), also known as CXCL8, is a chemokine best known for its role in neutrophil recruitment and has also been implicated in pain-related neuroimmune signaling [83]. IL-8 signals mainly through CXCR1 and CXCR2, which are expressed on neutrophils and other immune cells and have also been reported on sensory neurons [84]. In peripheral tissues, IL-8 contributes to innate immune activation by promoting leukocyte recruitment and local inflammatory signaling. Beyond its immune functions, IL-8 may exert pronociceptive effects by sensitizing peripheral nociceptors and promoting neuropeptide release [85]. These properties provide a plausible biological rationale for its involvement in pain, but do not, by themselves, establish IL-8 as a clinically useful biomarker.

At present, the most informative human data on IL-8 in CLBP come from the study by Rosenström et al., who measured IL-8 in CSF, serum, and disc tissue in patients with LDH and DDD. They reported significantly elevated IL-8 levels in the CSF of LDH patients compared with controls, with CSF concentrations correlating positively with pain intensity in males. In contrast, serum IL-8 levels showed the opposite pattern, being lower in patients than in controls and showing no association with pain [43].

This divergent pattern, characterized by elevated levels in CSF but reduced concentrations in serum, provides a particularly clear illustration of compartmentalized neuroinflammation. It suggests that IL-8 elevation primarily reflects inflammatory processes within the CNS rather than systemic inflammation [43]. The increased CSF IL-8 levels likely arise from a combination of sources, including activated spinal glia, infiltrating immune cells, and possibly retrograde transport from inflamed nerve roots or disc tissue [39].

The pathophysiological relevance of CSF IL-8 is further supported by its association with clinical pain measures. Higher CSF IL-8 levels were associated with greater back pain severity in males with LDH [43]. This sex-specific pattern mirrors observations reported for other neuroinflammatory biomarkers and may reflect sex-related differences in immune–pain interactions [82].

From a mechanistic perspective, elevated CSF IL-8 could contribute to pain through several complementary pathways. IL-8 may directly increase the excitability of spinal dorsal horn neurons [86] and promote microglial activation and chemotaxis, thereby amplifying neuroinflammatory signaling cascades [39]. In addition, IL-8 can increase vascular permeability and disrupt the blood–spinal cord barrier, facilitating the entry of peripheral immune cells into the CNS [87].

Despite its conceptual and mechanistic interest, the clinical utility of IL-8 as a biomarker is currently constrained by practical and methodological limitations. CSF sampling requires lumbar puncture, an invasive procedure that is not routinely performed in patients with CLBP outside research settings [88]. Accordingly, IL-8 should currently be regarded as a compartment-specific research marker that provides insight into neuroinflammatory mechanisms, rather than as a broadly applicable clinical biomarker. More generally, this example reinforces the principle that, for neuroinflammatory processes, measurements obtained within CNS compartments may be necessary to capture the most relevant pathophysiological signals [89].

### 4.3. Systemic Cytokines: IL-6, TNF-α and CRP

IL-6, TNF-α, and CRP are the most extensively investigated systemic inflammatory biomarkers in CLBP [79,90]. These mediators are biologically relevant to musculoskeletal inflammation and have been implicated in neuroimmune signaling pathways that may contribute to pain chronification [35].

IL-6 and TNF-α are key regulators of innate immune activation and are expressed within intervertebral disc and spinal tissues under inflammatory conditions [41]. Experimental studies demonstrate that both cytokines can enhance nociceptive transmission, promote synaptic excitability, and facilitate glial activation within the spinal cord [10,35]. Through these mechanisms, they represent biologically plausible contributors to CS in the context of persistent axial low back pain, including patients with degenerative disc changes in whom persistent pain mechanisms may extend beyond structural pathology.

In clinical cohorts of patients with CLBP, circulating IL-6 and TNF-α levels have been variably reported to be elevated compared with controls [60,90]. Case–control studies have de-scribed modest increases in systemic pro-inflammatory cytokines, although effect sizes are generally small and associations with pain intensity or disability inconsistent [90]. Longitudinal analyses in CLBP populations further suggest that persistent elevations of certain inflammatory mediators may be associated with less favorable clinical trajectories, though findings remain heterogeneous across independent cohorts [81].

CRP, as a downstream acute-phase reactant induced by IL-6 signaling, has likewise been examined in chronic axial low back pain populations. While some studies report mildly elevated CRP levels, differences are typically modest and influenced by age, adiposity, metabolic status, and comorbid conditions [53].

Collectively, these findings indicate that systemic inflammatory cytokines may reflect ongoing peripheral inflammatory activity and sustained nociceptive input in CLBP. However, circulating IL-6, TNF-α, and CRP provide only indirect and non-specific insight into central neuroinflammatory processes when compared with compartment-specific biomarkers. Their broader methodological and translational implications are further addressed in Section 5.

### 4.4. Emerging Neuroinflammatory and Metabolic Biomarkers

Beyond the biomarkers discussed above, several additional candidates have been proposed based on proteomic and high-throughput profiling approaches, including nucleoside diphosphate kinase A, glutathione S-transferase Pi (GSTPi), and interleukin-15 (IL-15) [91,92]. While these molecules are of scientific interest, the current evidence supporting their relevance in CLBP remains strictly exploratory.

A major limitation of these candidates is that they originate almost exclusively from discovery-phase studies and lack independent replication in well-characterized cohorts [93]. It is well recognized that a substantial proportion of initial biomarker signals identified through high-throughput screening fail to reproduce in subsequent studies [94]. Moreover, for most of these markers, there is currently insufficient mechanistic evidence linking them convincingly to established neurobiological pathways of pain chronification or CS.

Accordingly, at present, these emerging candidates should be regarded as hypothesis-generating signals rather than as validated or near-clinical biomarkers. Substantial additional work, including independent replication, longitudinal validation, and mechanistic characterization, will be required before any meaningful consideration of clinical translation.

## 5. Limitations of Systemic Inflammatory Biomarkers in CLBP

A central limitation of blood-based inflammatory biomarkers in CLBP is the compartmentalized nature of neuroinflammation [43,48]. Inflammatory processes relevant to CS may be confined to the spinal cord, nerve roots, or CSF and therefore may not be reflected in peripheral circulation due to the presence of the blood–brain and blood–spinal cord barriers [42,89]. As a result, systemic cytokine measurements may fail to capture the most pathophysiologically relevant processes [15,43]. This conceptual limitation is compounded by the lack of specificity of most circulating inflammatory markers, which are influenced by age, obesity, metabolic status, and multiple comorbid conditions [53,95,96]. Together, these factors explain why systemic biomarkers have shown limited and inconsistent utility in CLBP. These issues are well illustrated by three of the most frequently studied systemic inflammatory markers: IL-6, TNF-α, and C-reactive protein. IL-6 is a pleiotropic cytokine produced by multiple cell types, including immune cells, adipocytes, and skeletal muscle cells, and circulating levels are elevated in a wide range of conditions such as obesity, metabolic syndrome, and ageing [53,95,96]. In CLBP, studies assessing IL-6 have yielded conflicting results, with meta-analyses indicating only small or negligible effect sizes [79]. TNF-α, a central mediator of innate immune responses [97], shows similarly inconsistent behavior. Studies evaluating circulating TNF-α levels in CLBP have reported highly variable findings, and available clinical evidence indicates that the current data are insufficient to support TNF-α as a reliable biomarker in this context [79,81]. CRP, an acute-phase protein synthesized by the liver in response to IL-6 signaling [98], exhibits the same limitations. In CLBP, elevations in CRP are inconsistent and, when present, are often attributable to comorbid conditions rather than to pain-related inflammatory mechanisms per se [79]. Together, these observation underscore why systemic inflammatory markers have limited value for identifying neuroinflammatory pain mechanisms in CLBP and reinforce the need to prioritize CNS-compartment biomarkers or peripherally accessible markers with clear mechanistic specificity to neuroinflammatory pathways [15,48].

## 6. Clinical Implications and Translational Challenges

### 6.1. Biomarker-Based Phenotyping and Central Sensitization Results

The long-term objective of neuroinflammatory biomarker research is to enable precision medicine approaches in CLBP by matching patients to mechanism-targeted therapies based on their underlying pathophysiology. In this context, biomarker-based phenotyping aims to identify patients in whom pain is predominantly driven by neuroinflammatory processes and CS, and who may therefore benefit from neuromodulatory or centrally acting anti-inflammatory interventions [4,10].

Achieving this goal requires integration of multiple sources of information, including clinical assessment, QST, psychological profiling, and molecular biomarker measurements [15]. Recent studies have suggested that machine learning and other data-driven approaches may help identify clinically meaningful patient subgroups based on such multidimensional datasets [99,100]. Rather than relying on binary classifications, contemporary models increasingly conceptualize neuroinflammatory involvement as a continuum, with patients occupying different positions along a spectrum of biological and clinical phenotypes [99,101].

The combination of biomarkers with QST appears particularly promising. While QST provides functional characterization of altered pain processing, biomarkers offer insight into the underlying molecular and cellular mechanisms [102]. Concordance between QST profiles and neuroinflammatory biomarker signatures would provide convergent evidence for the presence of CS and could strengthen confidence in mechanism-based patient stratification [15].

Despite this conceptual appeal, major barriers currently limit clinical implementation. Validated cutoff thresholds for defining biomarker positivity are lacking, and the cost and limited availability of advanced biomarker technologies constrain scalability in routine practice [103,104]. Moreover, the absence of well-established mechanism-targeted therapies means that even accurate phenotyping may not yet translate into improved clinical outcomes without corresponding therapeutic options [15].

### 6.2. Methodological and Clinical Barriers

A number of methodological challenges continue to hinder progress in neuroinflammatory biomarker research in CLBP. One major issue is the lack of standardization across studies, with substantial variability in sample types, collection protocols, and assay platforms contributing to inconsistent and difficult-to-compare results [94,105]. In addition, many studies are limited by small sample sizes, reducing statistical power and increasing susceptibility to false-positive findings [94,106].

Most available data are derived from cross-sectional study designs, in which biomarkers are measured at a single time point, precluding meaningful assessment of temporal dynamics or causal relationships between neuroinflammation and pain. This limitation is compounded by the marked phenotypic and mechanistic heterogeneity of CLBP, as analyses of unselected patient cohorts are likely to dilute signals arising from specific neuroinflammatory subgroups [2,15,99].

Confounding by comorbid conditions represents another important challenge. Patients with CLBP frequently present with obesity, metabolic disorders, cardiovascular disease, or mood disturbances, all of which can influence inflammatory biomarker levels independently of pain mechanisms [53]. Failure to adequately control for these factors can lead to spurious or misleading associations.

In addition to clinical comorbidities, biological sex may represent another important and insufficiently explored source of variability in neuroinflammatory biomarker findings. Several studies included in this review reported sex-dependent associations, particularly for chemokines involved in neuroimmune signaling. Elevated CSF IL-8 levels were associated with pain intensity predominantly in male patients, whereas MCP-1 showed stronger associations with pain sensitivity measures in female cohorts [43,44]. However, sex-stratified analyses were inconsistently performed across studies, and most investigations were not specifically designed or statistically powered to detect sex differences. Consequently, current evidence should be interpreted cautiously, and apparent sex-specific findings remain hypothesis-generating rather than definitive. These observations align with broader evidence indicating sex-related differences in immune–pain interactions [82]. Future biomarker studies should incorporate predefined sex-stratified analyses and adequate sample sizes to clarify whether neuroimmune mechanisms differ systematically between males and females in CLBP.

Practical constraints further limit translation into clinical practice. CSF sampling requires lumbar puncture, an invasive procedure with limited acceptability outside research settings, and TSPO-PET imaging remains expensive and restricted to specialized centers [88,107]. Finally, and perhaps most importantly, there is currently a lack of prospective clinical trials demonstrating that biomarker-guided treatment selection improves patient outcomes, representing a critical gap in the translational pathway [16].

## 7. Future Directions and Research Priorities

To move neuroinflammatory biomarkers from exploratory research tools to clinically useful decision-making aids, a coordinated and methodologically rigorous research agenda is required. A central priority is the development of multi-compartment and multi-marker biomarker panels, as the complexity of neuroinflammatory mechanisms makes it unlikely that single biomarkers will provide sufficient sensitivity or specificity. Future approaches should therefore integrate glial markers, chemokines, and epigenetic signatures into composite biomarker profiles [15].

Progress in this field will also require large-scale longitudinal cohort studies with repeated biomarker assessments, QST, and standardized clinical outcome measures. Such designs are essential to characterize temporal dynamics, assess predictive validity, and distinguish trait from state biomarkers [17]. Crucially, biomarker research must be linked to interventional studies. Randomized controlled trials that test mechanism-targeted therapies in biomarker-defined patient subgroups will be necessary to demonstrate true clinical utility and to establish biomarker-guided treatment strategies [3,15].

Emerging evidence for sex differences in biomarker–pain associations highlights the importance of systematically incorporating sex-stratified analyses into future studies, rather than treating sex as a mere covariate [108]. Another important priority is the integration of molecular biomarkers with functional phenotyping tools such as QST, as combining these approaches may enable the development of composite algorithms capable of more robust and clinically meaningful patient stratification.

Given the limited feasibility of routine CSF sampling, substantial effort should also be directed toward identifying peripherally accessible biomarkers that reliably reflect CNS-compartment neuroinflammatory processes, including blood-based surrogate markers that correlate with CSF signatures. Methodologically, future work should increasingly adopt epigenetic and multi-omic strategies, moving beyond single-gene approaches toward genome-wide or systems-level profiling to capture broader molecular patterns associated with pain chronicity and CS.

Ultimately, only through the integration of longitudinal, multi-modal, and interventional designs will neuroinflammatory biomarkers be able to transition from mechanistic research tools to clinically actionable instruments.

### Toward Clinically Actionable Biomarker Research

To move neuroinflammatory biomarkers closer to clinical application, future research should focus on pragmatic study frameworks that remain feasible within real-world clinical settings. Rather than relying on extensive or highly specialized biomarker panels, near-term studies may benefit from combining functional phenotyping with a limited number of accessible biological measures. For example, QST could be integrated with a small panel of blood-based inflammatory or neuroimmune markers and standardized clinical outcomes assessing pain intensity, disability, and psychosocial burden. In selected cohorts, CSF sampling or TSPO-PET imaging may be incorporated as mechanistic substudies to better understand how peripheral signals relate to CNS processes.

Importantly, progress in this field should be evaluated using clinically meaningful endpoints rather than solely demonstrating statistical group differences. Relevant indicators of success may include the identification of patient subgroups characterized by CS features, the ability to predict response to specific treatments, or improved prognostic stratification regarding pain persistence and recovery trajectories.

From a methodological perspective, longitudinal designs with repeated sampling will be essential to capture temporal dynamics and clarify causal relationships. Future studies would also benefit from replication cohorts, nested mechanistic analyses, and pre-registered analytical strategies to improve reproducibility and reduce the risk of false-positive findings. Collectively, these approaches may help bridge the current gap between mechanistic insight and clinically actionable biomarker use in CLBP.

## 8. Conclusions

Neuroinflammatory biomarkers represent a conceptual shift in the understanding of chronic low back pain, moving the field beyond purely symptom-based classifications toward a mechanism-oriented framework. Converging evidence from preclinical studies, neuroimaging, and multi-compartment human investigations supports a central role for glial activation, neuroimmune signaling, and CS in a clinically relevant subset of patients with CLBP.

Among current candidates, glial markers such as GFAP and TSPO provide strong mechanistic foundations, chemokines including MCP-1 and IL-8 highlight periphery-to-CNS inflammatory cross-talk, and BDNF DNA methylation emerges as a promising epigenetic marker linking molecular mechanisms to clinical and biopsychosocial dimensions of pain. In contrast, traditional systemic inflammatory markers have shown limited and inconsistent value, underscoring the importance of compartmentalized neuroinflammation.

Despite this progress, major translational gaps remain. Methodological limitations, lack of standardization, and—most critically—the absence of prospective biomarker-guided interventional trials currently prevent routine clinical implementation. Future advances will therefore depend on integrated, multi-modal approaches combining biomarkers with functional phenotyping and on rigorous validation in longitudinal and interventional studies.

Ultimately, the translation of neuroinflammatory biomarkers into clinical practice could enable a more precise and biologically grounded approach to CLBP, in which treatments are guided by underlying mechanisms rather than by symptoms alone.

## Figures and Tables

**Table 1:** Neuroinflammatory biomarkers in CLBP: biological compartments, types of evidence, and translational readiness.

Biomarker	Primary Compartment	Human Evidence (Key References)	Typical Direction	Key Confounders	Main Limitations	Level of Evidence	Translational Readiness
**GFAP**	CSF; peripheral blood (less specific)	Elevated in neuroinflammatory states; emerging data in CLBP-related cohorts [12,14,45]	↑ in CSF; blood findings inconsistent	Age; neurodegenerative disease; systemic inflammation; BBB integrity	Limited CLBP cohorts; low specificity in blood; small samples	Mechanistic + limited human data	Early translational; not ready for clinical use
**TSPO-PET**	CNS (molecular imaging)	Increased TSPO binding in chronic pain and CLBP imaging studies [46,47,48,49,50]	↑ TSPO signal	TSPO polymorphisms; medication; scanner variability	High cost; limited access; small cohorts	Direct human imaging evidence	Research tool only
**MCP-1 (CCL2)**	CSF; serum	Elevated in DDD/LDH cohorts; linked to neuroimmune activation [43,44,51,52]	↑ in CSF; variable in serum	BMI; smoking; metabolic status; inflammatory disease	Not CLBP-specific; systemic overlap	Human CSF + translational evidence	Potential in composite panels
**IL-8 (CXCL8)**	CSF; serum	Elevated in CSF in lumbar disc cohorts; sex-dependent findings [43,53]	↑ in CSF; inconsistent systemically	Sex; obesity; systemic inflammation	Heterogeneous findings; limited longitudinal data	Human CSF evidence	Exploratory
**BDNF DNA Methylation**	Peripheral blood DNA	Altered methylation in chronic pain and CS features [54,55,56]	Altered methylation (CpG dependent)	Psychological stress; depression; early life stress	Small cohorts; limited replication	Epigenetic association studies	Exploratory; requires validation
**IL-6/TNF-α/CRP**	Serum	Most studied systemic markers; weak/inconsistent associations [57,58,59,60]	Mild ↑ or inconsistent	Age; obesity; metabolic syndrome; smoking	Low specificity; reflect systemic inflammation	Observational clinical studies	Limited utility alone
**Emerging multi-omic candidates (e.g., NDKA, GSTPi, IL-15)**	Serum; proteomic panels	Discovery-phase studies; preliminary associations with CLBP phenotypes	Inconsistent	Unknown; cohort heterogeneity	Lack of replication; exploratory findings	Discovery-phase evidence	Hypothesis-generating

↑: upregulated.

## Data Availability

Data sharing is not applicable to this article as no new data were created or analyzed in this study.

## Abbreviations

The following abbreviations are used in this manuscript:

BDNF	Brain-Derived Neurotrophic Factor
CLBP	Chronic Low Back Pain
CNS	Central Nervous System
CS	Central Sensitization
CSF	Cerebrospinal Fluid
DDD	Degenerative Disc Disease
DNA	Deoxyribonucleic acid
GFAP	Glial Fibrillary Acidic Protein
GSTPi	Glutathione S-Transferase Pi
IL-8	Interleukin-8
IL-15	Interleukin-15
LDH	Lumbar Disc Herniation
MCP-1	Monocyte Chemoattractant Protein-1
NDKA	Nucleoside Diphosphate Kinase A
NMDA	N-Methyl-D-Aspartate
PET	Positron Emission Tomography
QST	Quantitative Sensory Testing
TNF-α	Tumor Necrosis Factor alpha
TSPO	Translocator Protein
